# Exploratory analysis of epilepsy biomarkers using untargeted metabolomics across multiple cohorts

**DOI:** 10.3389/fneur.2025.1684456

**Published:** 2025-12-01

**Authors:** Kaisa T. Oja, Mihkel Ilisson, Karit Reinson, Kai Muru, Tiia Reimand, Hedi Peterson, Dmytro Fishman, Toomas Haller, Jaanika Kronberg, Estonian Biobank Research Team, Monica H. Wojcik, Adam D. Kennedy, Laura Sommerville, Gregory Michelotti, Anne O’Donnell-Luria, Eve Õiglane-Shlik, Sander Pajusalu, Katrin Õunap

**Affiliations:** 1 Department of Genetics and Personalized Medicine, Institute of Clinical Medicine, University of Tartu, Tartu, Estonia; 2 Genetics and Personalized Medicine Clinic, Tartu University Hospital, Tartu, Estonia; 3 Institute of Computer Science, Faculty of Science and Technology, University of Tartu, Tartu, Estonia; 4 STACC OÜ, Tartu, Estonia; 5 Estonian Genome Centre, Institute of Genomics, University of Tartu, Tartu, Estonia; 6 Center for Mendelian Genomics, Broad Institute of MIT and Harvard, Cambridge, MA, United States; 7 Division of Genetics and Genomics, Department of Pediatrics, Boston Children’s Hospital, Boston, MA, United States; 8 Division of Newborn Medicine, Department of Pediatrics, Boston Children’s Hospital, Boston, MA, United States; 9 Metabolon, Morrisville, NC, United States; 10 Department of Pediatrics, Institute of Clinical Medicine, Faculty of Medicine, University of Tartu, Tartu, Estonia; 11 Children's Clinic, Tartu University Hospital, Tartu, Estonia

**Keywords:** epilepsy, metabolomics, lipidomics, phosphatidylcholines, exploratory

## Abstract

**Introduction:**

Epilepsy is a common central nervous system disorder characterized by abnormal brain electrical activity. Diagnosing epilepsy can be challenging, as it has various causes and different clinical manifestations. We aimed to analyze whether there are similarities in the metabolic profiles of patients with epilepsy despite different etiology, seizure frequency, seizure type, and patient age.

**Methods:**

Untargeted metabolomics analysis results were analyzed in three cohorts. The pediatric cohort (PED-C) consisted of 110 pediatric individuals with suspicion of a genetic disorder with unclear etiology; the adult cohort (AD-C) of 250 adults from the Estonian Biobank (EstBB), and the elderly cohort (ELD-C) of 583 adults ≥ 69 years from the EstBB. Epilepsy was diagnosed in 35, 11 and 26 cases, respectively.

**Results:**

Significant differences (FDR corrected *p*-value <0.05) were detected in the PED-C in eight metabolites, mainly for lipids. Restricting the analysis to individuals with epilepsy and without any changes in brain magnetic resonance imaging increased the number of significant metabolites to 16. Nine significantly altered lipids were found in the AD-C, mainly triacylglycerides (TAGs). In the ELD-C, significant changes in 20 metabolites, including multiple TAGs, were observed in the metabolic profile of participants with previously diagnosed epilepsy.

**Discussion:**

Several components of cell membranes were among the altered metabolites, indicating that cell membrane fluidity may have a significant role in the mechanism of epilepsy, and changes in lipid balance may indicate epilepsy. However, further studies are needed to evaluate whether untargeted metabolomics analysis could prove helpful in diagnosing epilepsy earlier.

## 1 Introduction

Epilepsy is a common central nervous system disorder characterized by abnormal brain electrical activity and defined as having at least two unprovoked seizures or one unprovoked seizure with a high probability of further seizures (1). An estimated 50 million individuals worldwide have been diagnosed with epilepsy (2). It occurs in adults and children, with the youngest and oldest age groups affected the most (3–6). In a recent epidemiological study of childhood epilepsy in Estonia, the overall incidence was 86.3 cases per 100,000 person-years (7). The leading cause of epilepsy differs by age groups with structural and genetic causes being more common in early-onset epilepsy and stroke and neoplasia in the elderly (5–7). In Estonia, the reported prevalence rate (PR) of childhood epilepsy is 3.6 per 1,000 (8), and the PR of epilepsy in the adult population is 5.3 per 1,000 population (9), which is comparable to prevalence rates reported in other developed countries (3). About a third of patients have drug-resistant epilepsy and poor seizure control (10). In 2017, misdiagnosis was estimated to be around 20% with many patients having psychogenic non-epileptic seizures or other types of episodes instead (11).

The classification system of epilepsy is complex and places great importance on ascertaining the etiology (12). Different metabolic disorders are associated with epilepsy and constitute a separate etiological group (9). Wang et al. showed that common metabolic features could be found in at least three types of seizures (13). Changes in glutamate, lactate, and citrate levels and disruptions in related biochemical pathways—such as amino acid metabolism and pathways linked to neurotransmitter and neurometabolic functions—have been associated with epilepsy (14). These include alanine, aspartate, and glutamate metabolism; glycine, serine, and threonine metabolism; glycerophospholipid metabolism; and arginine and proline metabolism (14). Recent studies have therefore focused on metabolomics to understand the pathophysiology of epilepsy and find new targets for antiepileptic drugs (AEDs) (13, 14). Metabolomics has been a leading laboratory method in small molecule biomarker discovery, and it complements other omics, including genomics. The expansion of diagnostic assays using new biomarkers, whether small molecular weight compounds or proteins, can increase the phenotypic resolution of many diseases. This can be accomplished through targeted panels of compounds or through clinical metabolomics, the analysis of the comprehensive metabolome of a sample. In addition to identifying the biomarkers of disease, researchers and clinicians need to identify and track the biomarkers of drug efficacy, toxicity and interactions.

Additionally, lipidomics, the measurement of the comprehensive set of molecules comprising lipids (e.g., sphingolipids, sphingomyelins, diacylglycerols, cholesterol esters, and the fatty acid compositions of each of these subclasses), can provide further insight into the biomarker profiles of disease. This is of specific interest for neurological diseases because lipids can play a central role in neural cell activation, maturation, and intracellular signaling processes (15–17). Changes in lipid distribution (especially glycerophospholipid and glycerolipid distribution) between the hippocampus and cortex, potentially leading to loss of membrane integrity and perturbations in synaptic signaling, have been described in mouse models of epilepsy (18). Three lipid metabolism pathways (glycerophospholipid metabolism, glycosylphosphatidylinositol-anchor biosynthesis, and glycerolipid metabolism) have also been connected to epileptic hippocampal tissue injury in rat models (19).

Given the ability of metabolomics to reveal deep phenotypic information on disease, common biochemical signatures in patients diagnosed with epilepsy could identify diagnostic biomarkers of epilepsy and advance our understanding of the mechanisms of disease activity, and reveal targets for therapeutic intervention. We hypothesize that a typical metabolic profile might help to reduce the misdiagnosis of epilepsy. The goal of this study was to determine whether there are similarities in the metabolic profiles of patients with epilepsy despite different etiology, seizure frequency, seizure type, and patient age. We aimed to look for metabolic profiles of more specific disease groups. To accomplish this, we compared untargeted metabolomics and lipidomics data between individuals with and without epilepsy in three cohorts and smaller subgroups.

## 2 Materials and methods

### 2.1 Subjects

We used data from three cohorts that varied in general characteristics (Table 1) based on different inclusion criteria. The pediatric cohort (PED-C) consisted of 110 pediatric patients who were suspected of having a monogenic neurodevelopmental disorder, but no clear disease etiology had been found. These subjects were enrolled in the Genetics and Personalized Medicine Clinic at Tartu University Hospital between 2016–2018 and 2019–2023. The adult cohort (AD-C) consisted of 250 adults (aged 20–64 years) from the Estonian Biobank (EstBB) (20), and the elderly cohort (ELD-C) consisted of 583 elderly participants (aged 69–79 years) from the EstBB (21). The EstBB is a volunteer-based biobank representing about 5% of the Estonian adult population (22). All participants from the EstBB were enrolled before 2011 and were selected for our study if they had metabolomics data available for analysis from previous projects.

**Table 1:** General characteristics of all three cohorts.

Characteristic	PED-C	AD-C	ELD-C
Cohort size	*n* = 110	*n* = 250	*n* = 583
Mean (range) age in years	7 (1.2–17)	39 (20–64)	73 (69–79)
Female (%)	41 (37.3)	122 (48.8)	407 (69.8)
Epilepsy diagnosis (%)	35 (31.8)	11 (4.4)	26 (4.5)
Mean (range) age of epilepsy onset in years	4.1 (0.1–15)	28.8 (5–64)	79.4 (66–90)
Epilepsy onset before sampling (%)	31 (88.6)	7 (63.6)	5 (19.2)

The mean age of onset and percentage of epilepsy onset before sampling were calculated for epilepsy-positive subgroups in each cohort. All patients were of European ancestry.

The PED-C was a subset of a cohort of 131 unsolved patients with suspected genetic disorders without molecular diagnosis (ORPHA:616874 “rare disorder without a determined diagnosis after full investigation”) who had agreed to participate in a multi-omics study and had given a sample for metabolomics research. Informed consent was obtained from all subjects involved in the study or their legal guardians. Participants aged 7–17 were given an additional age-appropriate informed consent form. We excluded 21 samples from this study due to ambiguous records regarding epilepsy, age >18 years, or investigation regarding a specific alternate project. One sample was excluded because the patient had enteroviral encephalomyelitis, and one sample had been collected shortly before the patient passed away. Among the 110 individuals included, one case was a confirmed O-linked N-acetylglucosamine transferase glycosylation (OGT-CDG) defect, and one had xanthinuria.

The AD-C consisted of participants from a larger cohort selected to represent body-mass-index (BMI) extremes and their randomly matched controls (20). Individuals with pregnancy, anorexia, or a known wasting illness were excluded (20). The original cohort consisted of 300 individuals, with an equal number of the lean, obese, and general population from the EstBB (20, 23). Our subset included the 100 population controls, 73 lean and 77 obese individuals whose metabolomics data was available.

ELD-C included participants between 69 and 79 years old at the time of sampling. Individuals with a history of hypertensive heart disease, diabetes, coronary artery disease, cancer, chronic obstructive pulmonary disease, stroke, or Alzheimer’s disease were excluded (21). The two EstBB cohorts did not overlap (20).

We requested access to the metabolomics and lipidomics data of the AD-C and ELD-C, which had already been analyzed in separate projects (20, 21, 23). Individual-level data analysis in the EstBB was carried out under approval no 1.1–12/3749 from the Estonian Committee on Bioethics and Human Research (Estonian Ministry of Social Affairs), using data according to release application “R04” from the Estonian Biobank. Before participating in the EstBB, all individuals gave informed consent for their biological samples to be used for future studies.

### 2.2 Clinical data collection

We analyzed electronic medical records from the PED-C, which were made available by the Genetics and Personalized Medicine Clinic and the Children’s Clinic of Tartu University Hospital. Information on the patients’ intellect, behavior, development, growth, facial features, epilepsy, brain magnetic resonance imaging (MRI), and movement was collected in addition to general characteristics, including age, sex, medications used at the time of sampling, and perinatal history. For the AD-C and ELD-C, information from the Biobank was obtained on sex, age, body mass index (BMI), diagnosis of dyslipidemia and epilepsy, age at epilepsy diagnosis, treatment with antiepileptic drugs (Anatomical Therapeutic Chemical (ATC) code N03A) at the time of sampling. Dyslipidemia was defined as the diagnosis of E78 (disorders of lipoprotein metabolism and other lipidemias) according to the International Classification of Diseases (ICD-10), and epilepsy as G40 (epilepsy) or G41 (status epilepticus) with all their subgroups (24). Any aberrations on the brain MRIs were classified as a brain structural anomaly in the PED-C.

### 2.3 Metabolomics analyses

Four ml of EDTA plasma was collected from all patients in PED-C for metabolomics and lipidomics analysis. Fasting before sample collection was not required. The collected plasma samples were frozen (at ≤ − 20 °C, no thawing) and shipped to Metabolon, Inc. (USA), where untargeted metabolomics and lipidomics analysis was performed using the Meta IMD test (26). This Laboratory-Developed Test (LDT), performed in a CLIA-certified laboratory, detects over 800 small molecules that range from 50 to 1,500 Daltons. The test uses four different methods of Ultra-High Performance Liquid Chromatography (UHPLC) instruments paired with Mass Spectrometry (UHPLC/ MS). Detected molecules were identified by comparison to a biochemical reference library, with unique biochemical entries characterized by accurate molecular mass, including information on any adductation, in source fragmentation, and/or polymerization (typically dimers and trimers) and retention time/index on the chromatography columns. The results were reported as z-scores, the number of standard deviations from the mean for each metabolite, which were calculated using a reference cohort of 866 healthy pediatric samples. We received data for 499 known metabolites.

In the AD-C, metabolite profiling had also been done using four liquid chromatography-mass spectrometry (LC–MS) methods (20). In the original dataset of 300 individuals, the mean fasting time was 7.4 h (+/− 3.3 SD) (20). We received data for 327 known metabolites and 17,774 unknown compounds.

The ELD-C non-fasting plasma samples (mean fasting time was 3.04 h +/− 2.80 SD; however, 200 samples were missing this data) were collected and stored in liquid nitrogen before shipment to the Broad Institute (Cambridge, MA, USA) for metabolomic profiling using LC–MS. (21) The metabolites were measured with four different methods described in detail by Esko et al. (27). We received data for 585 known metabolites and >19,000 unknown compounds. The methods used for the AD-C and ELD-C have been described in detail by Hsu et al. (20).

### 2.4 Statistical testing

The computational and programming tasks were performed with Python 3.7. Pandas, Numpy, Sklearn, Scipy libraries, and Microsoft Excel were used for data handling and statistical testing.

GraphPad Prism 8 was used for data visualization (volcano plots).

Statistical tests were done separately for each cohort. The untargeted metabolomic analyses were not compared between cohorts due to differences in the experimental setup used for each cohort. Unidentified compounds were excluded from all analyses.

Univariate statistical tests were performed to investigate whether a particular metabolite could be associated with epilepsy. Kruskal-Wallis tests were performed, and after confirming the normal metabolite distributions for both epilepsy positive and negative classes by Shapiro–Wilk test (*p* > 0.05), Welch t-tests were also performed. Extracted *p*-values for each test were corrected with FDR using the Benjamini-Hochberg approach. A significance threshold of α = 0.05 was used to interpret results. Z-score difference of observed groups and Cohen’s d were calculated to assess the effect sizes of statistically significant metabolites. Additionally, power calculations were performed for each statistically significant metabolite to monitor the statistical power of univariate testing.

Common confounders (age, gender, treatment, AED use, and other factors depending on the cohort) were analyzed using multilinear regression analysis (script in Supplementary materials). Univariate test effect sizes and multilinear analysis beta-values were compared to evaluate the effect of confounders.

Correlation between metabolite concentrations was evaluated by calculating Pearson correlation coefficients *r*.

## 3 Results

### 3.1 Clinical features

The composition of the PED-C cohort is outlined in Table 1. The male-to-female ratio was 1.7:1, and 35/110 (31.8%) patients had been diagnosed with epilepsy, although in four cases (4/35, 11.4%), the disease onset was after metabolomics sampling (incident cases). The different epilepsy diagnosis codes are shown in Supplementary Table S1; several patients had been diagnosed with both focal and generalized epilepsy throughout their disease history. In eight cases, the diagnosis “epileptic seizures related to external causes” (G40.5) was used. We collected data about the age of onset, clinical characteristics, electroencephalogram (EEG), brain imaging findings, and treatment at sampling for patients diagnosed with epilepsy (Supplementary Table S1). Considering the different severity of epilepsy, the number of antiepileptic drugs (AEDs) varied from zero to four. At the time of sampling, nine (25.7%) individuals were not taking any AEDs, eleven (31.4%) were taking one AED, ten (28.6%) were taking two AEDs, and five (14.3%) had a combination of three or more medications. The most commonly used AEDs were levetiracetam (nine individuals) and valproic acid (nine individuals). Other medications included cannabidiol, carbamazepine, clonazepam, ethosuximide, lamotrigine, oxcarbazepine, phenobarbital, phenytoin, sulthiame, topiramate, and vigabatrin. None of the patients was on a ketogenic diet. The most common comorbidities in the PED-C were developmental language disorder (84/110, 76.4%), dysmorphic features (62/110, 56.4%), and intellectual disability (61/110, 55.5%). We compared the occurrence of various clinical features in individuals with and without epilepsy in the PED-C cohort, as shown in Table 2, and a more detailed distribution is shown in Supplementary Table S2. The frequency of brain structural anomalies was significantly higher (*p* = 0.0001) in the epilepsy subgroup.

**Table 2:** Characteristics of epilepsy positive and negative subgroups in PED-C.

Clinical features in the PED-C	Individuals with epilepsy (*n* = 35)	Individuals without epilepsy (*n* = 75)	*p*-value
Mean age (range)	8.5 (1.2–17)	7.9 (2–16.9)	0.51
Female (%)	17 (48.6)	24 (32)	0.14
Brain structural anomalies (%)	24 (68.6)	21 (28)	**0.0001**
Neuromuscular and/or extrapyramidal symptoms (%)	20 (57.1)	30 (40)	0.10
Intellectual disability (%)	23 (65.7)	38 (50.7)	0.15
Autism spectrum disorder (%)	11 (32.4)	36 (48)	0.15
Abnormal growth (%)	13 (37.1)	23 (30.7)	0.52
Microcephaly (%)	6 (17.1)	9 (12)	0.55
Dysmorphic features (%)	19 (54.3)	43 (57.3)	0.84
Developmental delay (%)	27 (79.4)	60 (80)	1

Fisher’s exact or Chi-square test was used for categorical variables, and Welch *t*-test was used for continuous variables. Statistical significance was set at *p* < 0.05 and is shown in bold.

In the AD-C, epilepsy prevalence was 2.8% (7/250) at the time of sampling, which is higher than the age-adjusted (to the 1970 US population) prevalence rate of epilepsy in the Estonian adult population (9). Four additional cases had been diagnosed at the time of data retrieval, and the final prevalence was 4.4% in the AD-C. At the time of metabolome sampling, three participants with epilepsy were receiving treatment with antiepileptic drugs (respectively carbamazepine, primidone, and topiramate, Supplementary Table S3). All three of these patients had disease onset in childhood. Dyslipidemia diagnosis was present in 65 cases (26%) in the AD-C, four of them belonged to the epilepsy subgroup (4/11; 36.4%). The mean age, sex distribution, and BMI did not differ statistically significantly between epilepsy positive and negative subgroups in the AD-C (Table 3).

**Table 3:** Characteristics of the AD-C and ELD-C subgroups based on the presence of epilepsy.

Clinical features	AD-C			ELD-C		
	Epilepsy (*n* = 11)	No epilepsy (*n* = 239)	*p*-value	Epilepsy (*n* = 26)	No epilepsy (*n* = 557)	*p*-value
Mean age (range)	35 (20–56)	39 (20–64)	0.36	73 (70–78)	73 (69–79)	0.61
Female (%)	3 (27.3)	119 (49.8)	0.25	14 (53.8)	393 (70.5)	0.11
Median (IQR) BMI	24.3 (7.5)	26.4 (17.7)	0.36	27.7 (7.5)	27.0 (5.4)	0.97
Dyslipidemia diagnosis (%)	4 (36.4)	61 (25.5)	0.48	9 (34.6)	214 (38.4)	0.83

Welch t-test was used for age, Chi-square test for sex, Wilcoxon rank sum test with continuity correction for BMI, and Fisher’s exact test for dyslipidemia diagnosis. Statistical significance was defined as *p* < 0.05.

In the ELD-C, epilepsy had been diagnosed in 0.9% at the time of sampling, and this is similar to the other reports in this age group in developed countries (28). One individual was receiving treatment with clonazepam. At the time of data retrieval, 21 additional cases had been diagnosed. Dyslipidemia had been diagnosed in 223/583 cases (38.3%) in the ELD-C, nine of whom belonged to the epilepsy subgroup, making up approximately one third of the individuals with epilepsy (9/26, 34.6%). The mean age, sex distribution, and BMI did not differ statistically significantly between epilepsy positive and negative subgroups (Table 3). In brief, although the three cohorts were dissimilar and had different characteristics available for analysis, the frequency of brain structural anomalies in PED-C was the only statistically significant difference between epilepsy positive and negative subgroups in any of the cohorts. In AD-C and ELD-C, there was no available information regarding brain structure.

### 3.2 Metabolomics results

We compared the metabolic profiles of individuals with and without epilepsy in the three cohorts and specific subgroups within those cohorts, as shown in Table 4 for PED-C; Table 5 for AD-C and ELD-C.

**Table 4:** Summary of significantly altered metabolites in PED-C and its subgroups.

Compared subgroups	Significantly altered metabolites or metabolite classes	Number of metabolites
Whole PED-C cohort (*n* = 110)		
EP+ (*n* = 35) vs. EP− (*n* = 75)	1 dihydrosphingomyelin, eicosanedioate (C20-DC), kynurenate, 1 plasmalogen, 2 xenobiotics	↓ (*n* = 6)
	2 PCs	↑ (*n* = 2)
Brain anomalies present (*n* = 45)		
EP+ (*n* = 24) vs. EP− (*n* = 21)	No significantly altered metabolites	-
No known brain anomalies (*n* = 63)		
EP+ (*n* = 11) vs. EP− (*n* = 52)	3-hydroxy-3-methylglutarate, aconitate cis or trans, arabonate/xylonate, aspartate, beta-citrylglutamate, 3 dihydrosphingomyelins, N-acetylneuraminate, orotidine, vanillylmandelate	↓ (*n* = 11)
	2 acyl carnitines (medium chain), 1 androgenic steroid, 2 PCs	↑ (*n* = 5)
Treated (*n* = 101)		
EP+ (*n* = 26) vs. EP− (*n* = 75)	1 dihydrosphingomyelin, eicosanedioate (C20-DC), kynurenate, 1 plasmalogen, vanillylmandelate, 2 xenobiotics	↓ (*n* = 7)
	1 androgenic steroid, 2 PCs	↑ (*n* = 3)
Prevalent cases (*n* = 107)		
EP+ (*n* = 31) vs. EP− (*n* = 76)	7-methylguanine, 4-vinylphenol sulfate, 2 acyl carnitines, beta-citrylglutamate, 2 dihydrosphingomyelins, eicosanedioate (C20-DC), erythritol, erythronate*, 1 GPE, N6, N6, N6-trimethyllysine, O-sulfo-tyrosine, 1 PC, 5 plasmalogens, quinolinate, 4 tryptophane metabolites, vanillylmandelate (VMA)	↓ (*n* = 25)
	4 PCs	↑ (*n* = 4)

EP+, epilepsy positive; EP−, epilepsy negative; GPE, glycerophosphatidylethanolamine; PC, phosphatidylcholine; ↓, decreased in epilepsy positive subgroup; ↑, increased in epilepsy positive subgroup.

**Table 5:** Summary of significantly altered metabolites in AD-C and ELD-C and their subgroups.

Cohort	Compared subgroups	Significantly altered metabolites or metabolite classes	Number of metabolites
AD-C	Whole cohort (*n* = 250)		
	EP+ (*n* = 11) vs. EP− (*n* = 239)	1 PE, 8 TAGs	↓ (*n* = 9)
	Prevalent cases (*n* = 246)		
	EP+ (*n* = 7) vs. EP− (*n* = 239)	alanine, 1 CE, 2 DAGs, glycolithocholate, glycoursodeoxycholate, 3 PCs, 1 PC plasmalogen A, 1 PE, 1 PE plasmalogen, 1 PS, succinate, 14 TAGs	↓ (*n* = 28)
ELD-C	Whole cohort (*n* = 583)		
	EP+ (*n* = 26) vs. EP− (*n* = 557)	Hydrochlorothiazide	↓ (*n* = 1)
	Prevalent cases (*n* = 562)		
	EP+ (*n* = 5) vs. EP− (*n* = 557)	4-guanidinobutanoic acid, 4-pyridoxate, alpha-muricholate, anserine, C24:1 ceramide (d18:1), cortisone, cotinine, hydrochlorothiazide, hydrocinnamic acid, thiamine, 10 TAGs	↓ (*n* = 20)
	Incident cases (*n* = 578)		
	EP+(*n* = 21) vs. EP− (*n* = 557)	C4-OH carnitine, hydrochlorothiazide	↓ (*n* = 2)

EP+, epilepsy positive; EP−, epilepsy negative; TAG, triacylglyceride; PE, phosphatidylethanolamine; DAG, diacylglyceride; PC, phosphatidylcholine; PS, phosphatidylserine; CE, cholesteryl ester; ↓, decreased in epilepsy positive subgroup; ↑, increased in epilepsy positive subgroup.

**Figure 1:**
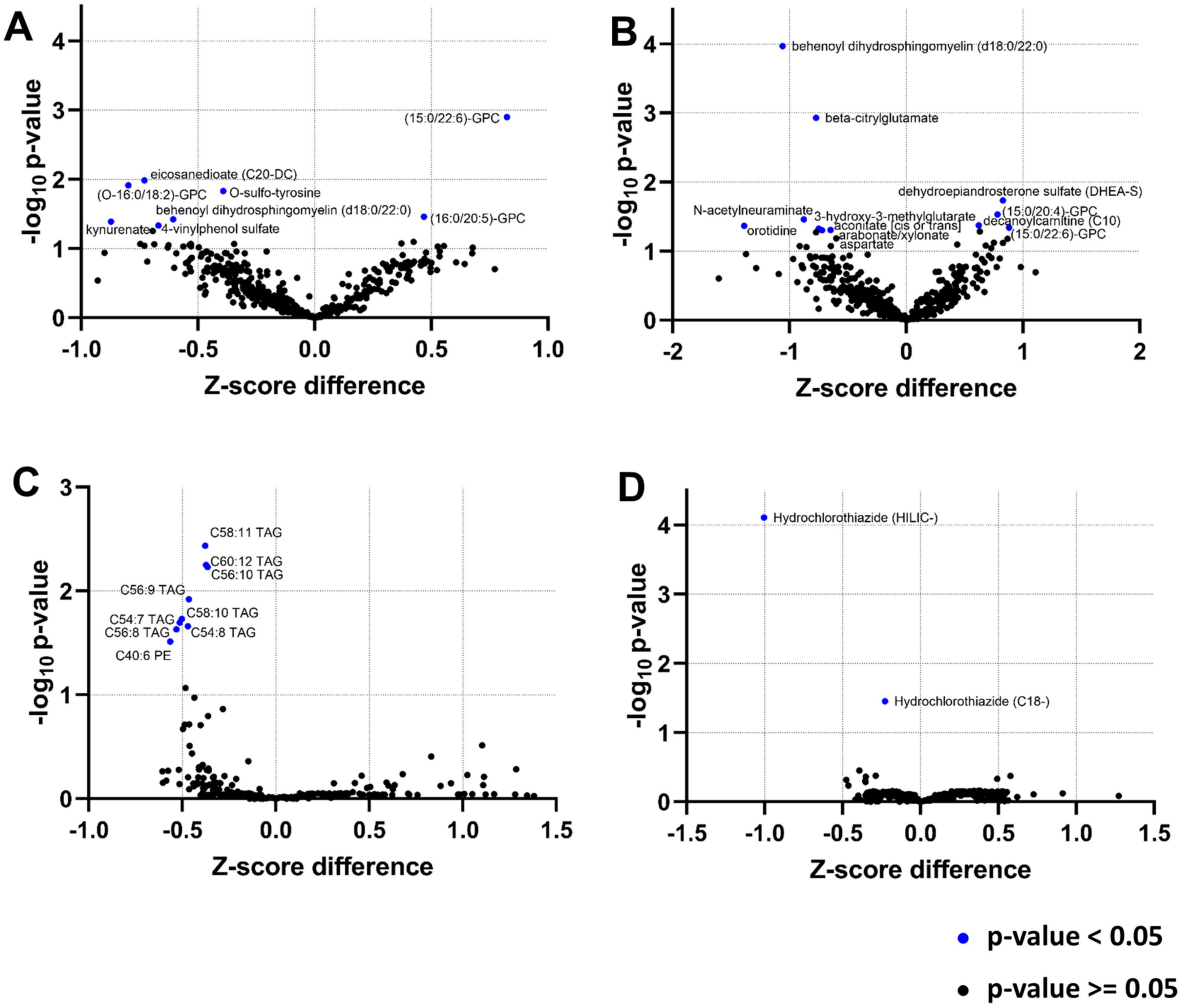
Metabolite volcano plots in different cohorts: **(A)** PED-C; **(B)** PED-C subgroup without brain anomalies; **(C)** AD-C; **(D)** ELD-C. The statistically significant metabolites are labelled and indicated with blue dots.

In the PED-C, the levels of eight metabolites out of 499 were significantly different (FDR-corrected *p*-value <0.05) between patients with and without epilepsy (Figure 1A). These included five lipids [three phosphatidylcholines (PC)], two xenobiotics, and kynurenate (a metabolite of tryptophan metabolism) (Table 4; Supplementary Table S4). Multilinear regression analysis indicated the possibility of confounding factors (Supplementary Tables S4, S5). Considering this and the availability of clinical data for this cohort, we performed additional analysis in different subgroups focusing on the presence of brain structural anomalies and AED treatment at the time of sampling.

Firstly, we analyzed the metabolic profile of individuals without brain structural anomalies (*n* = 63) by comparing the results of individuals with and without epilepsy within this subgroup, revealing 16 significant metabolites (Supplementary Table S4; Figure 1B). Five of these (sphingomyelin (d18:0/18:0, d19:0/17:0), sphingomyelin (d18:0/20:0, d16:0/22:0), behenoyl dihydrosphingomyelin (d18:0/22:0), aspartate, and orotidine) had a < 20% change in effect size compared to univariate testing, indicating that confounding factors did not strongly impact these. Several metabolites (beta-citrylglutamate, N-acetylneuraminate, 3-hydroxy-3-methylglutarate, arabonate/xylonate, aconitate [cis or trans]) showed a negative change in effect, which might be caused by a confounding factor that lessens the actual impact. For 3-hydroxy-3-methylglutarate and beta-citrylglutamate, this masking factor is likely to be AED use (Supplementary Table S5).

**Figure 2:**
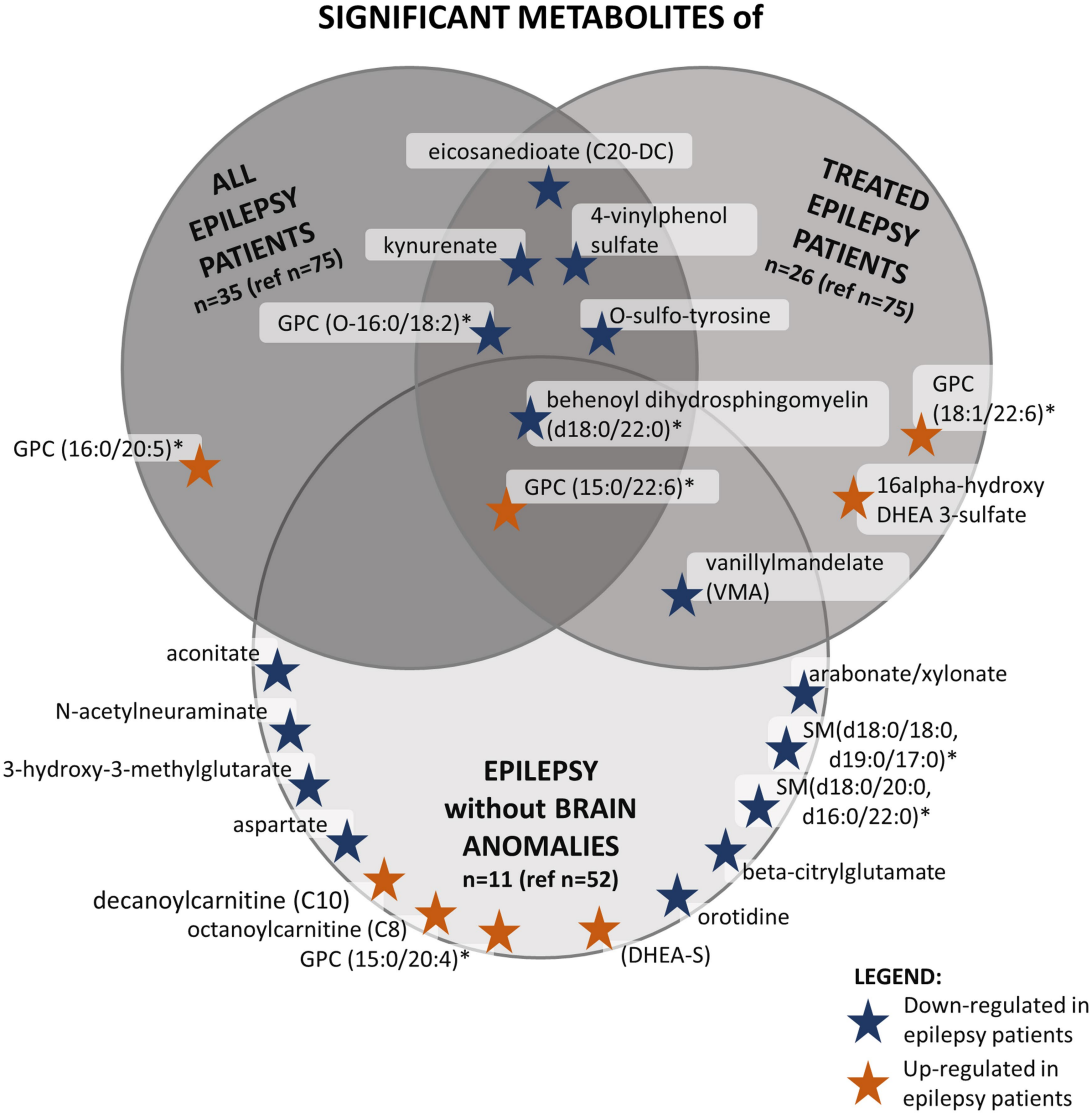
This figure shows the significantly altered metabolites in three different analyses in the PED-C subgroups and the overlap between them. The dark gray circle in the top left corner shows significant metabolites found when comparing all epilepsy patients to patients without epilepsy in PED-C. The medium gray circle in the top right corner shows metabolites that are significantly altered when comparing epilepsy patients taking AEDs to patients without epilepsy. The light gray circle at the bottom contains significantly altered metabolites when comparing individuals with and without epilepsy within the no brain anomalies subgroup. *Indicates a compound that has not been confirmed based on a standard, but we are confident in its identity (Not Tier 1); GPC, glycerophosphatidylcholine; SM, sphingomyelin.

**Figure 3:**
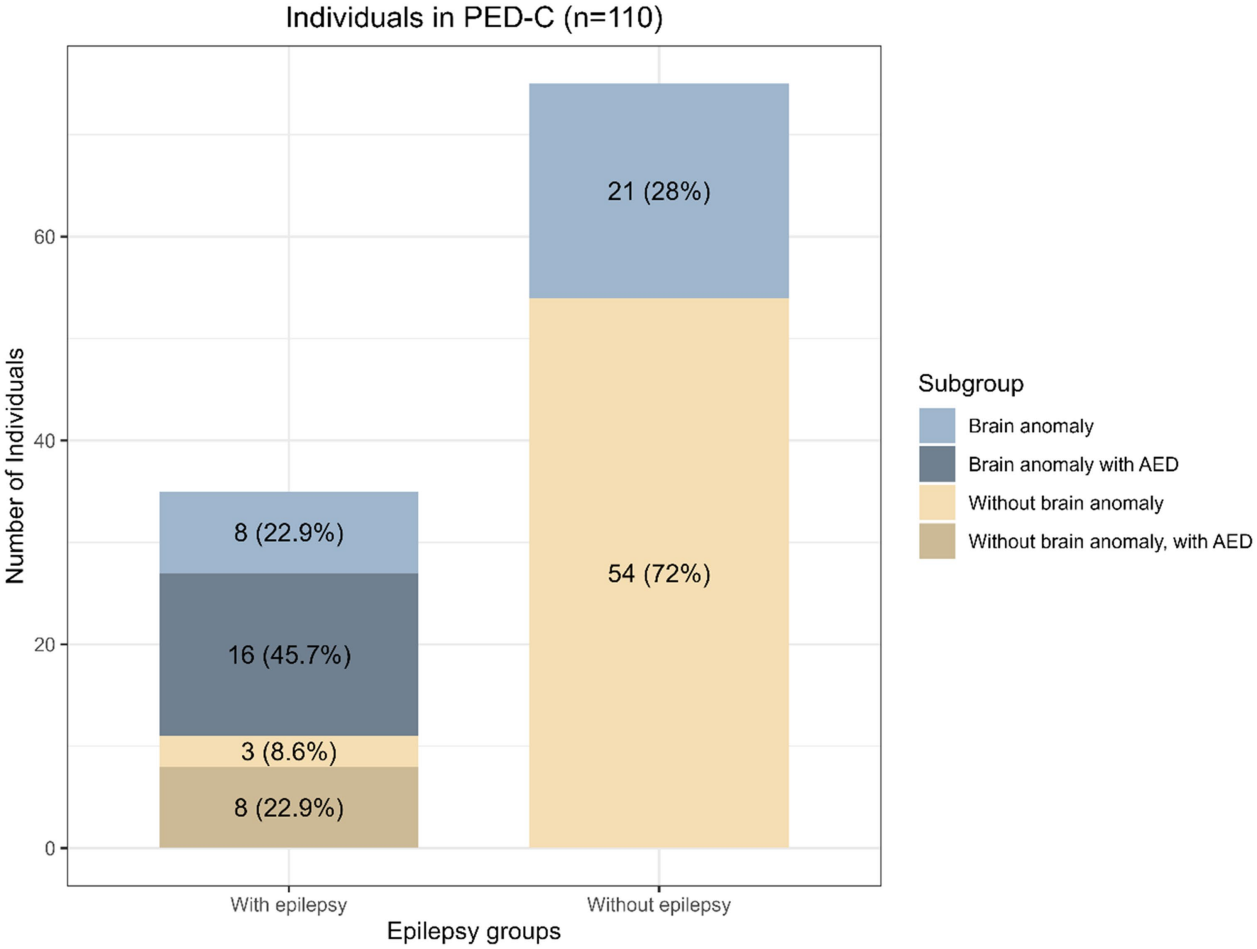
The distribution of phenotypes in PED-C. The blue-gray tones indicate subgroups of individuals with brain structural anomalies and the tan tones indicate individuals without. The proportion of individuals with brain structural anomalies is higher in the epilepsy group.

Secondly, we analyzed separately patients with epilepsy receiving AED treatment (*n* = 26) and patients without epilepsy (*n* = 75). Ten metabolites were significantly changed (Figure 2). In the not-treated subgroup (*n* = 84, including nine individuals with epilepsy), no statistically significant differences were found between individuals with and without epilepsy. However, one-third (3/9, 33.3%) of those individuals in the epilepsy subgroup had disease onset after sampling, and about two-thirds (7/9, 77.8%) had a brain structural anomaly as well. The overlap of individuals belonging to these subgroups is illustrated in Figure 3, and the overlap of significantly differing metabolites in these analyses is illustrated in Figure 2.

Additionally, since brain structural anomalies were significantly more common in the epilepsy subgroup, we also compared individuals with brain structural anomalies to individuals without brain structural anomalies in the epilepsy subgroup (*n* = 35) and the PED-C cohort in general (*n* = 109). Within the epilepsy subgroup, two sphingomyelins had significantly higher plasma levels, and lauroylcarnitine had a lower level in the individuals with brain structural anomalies (*n* = 24) compared to individuals without brain structural anomalies (*n* = 11) (Supplementary Table S4). In the PED-C, comparing all individuals with and without brain structural anomalies revealed that individuals without brain structural anomalies had lower levels of bilirubin and 1-palmityl-2-linoleoyl-GPC (O-16:0/18:2)*.

Lastly, we analyzed prevalent cases (epilepsy onset before sampling) versus individuals without epilepsy, adding another 21 significantly altered metabolites to the previously detected eight. Three out of four incident cases removed from this analysis had brain structural anomalies.

In the AD-C, nine significantly changed metabolites were found (Supplementary Table S6; Figure 1C). These were mainly triacylglycerides (TAG), precursors in the glycerophosphatidylcholine (GPC) synthesis pathway. We also detected one altered phosphatidylethanolamine in this cohort (Table 5). BMI had a strong positive correlation with the altered TAGs (Supplementary Table S7), but the change in effect size was low (Supplementary Table S6). Metabolite correlation analysis showed that the TAGs are strongly correlated with each other (Supplementary Table S8). The only significantly altered metabolite in the ELD-C was hydrochlorothiazide (Supplementary Table S9; Figure 1D), with an average z-score of −0.2145 (corrected *p*-value 0.0001).

In the EstBB cohorts, all the changed metabolite concentrations were lower than average in the epilepsy subgroup, but in the PED-C, some concentrations were higher than average (Tables 4, 5, Figures 1, 2).

In the ELD-C, we performed an additional analysis by dividing the epilepsy-positive subgroup into two subsets based on epilepsy onset before (prevalent cases) or after (incident cases) metabolomics sampling. C4-OH carnitine and hydrochlorothiazide were significantly altered in incident cases relative to non-epileptics. We observed significant changes in 20 metabolites, including multiple TAGs, in prevalent cases relative to non-epileptics. Three individuals out of the five prevalent cases (60%) had been diagnosed with dyslipidemia, while in the individuals without epilepsy, it had been diagnosed in 214 subjects (38%).

Finally, we compared the prevalent epilepsy cases in AD-C to epilepsy negative controls. Eighteen metabolites were significantly altered and included mainly triacylglycerols, diacylglycerols, and glycerophospholipids.

## 4 Discussion

We compared the metabolic profiles of individuals with and without epilepsy in three cohorts. We did not observe a specific metabolic profile in a heterogeneous epilepsy cohort of individuals of different ages, disease etiology, and comorbidities.

Interestingly, the additional analyses in PED-C indicate that brain structural anomalies have a substantial effect on the metabolic profile, as there were no significant differences between individuals with and without epilepsy within this subgroup. At the same time, comparing individuals with and without brain structural anomalies revealed only two statistically significant metabolites. Therefore, we focused on the 16 significantly changed metabolites (Figure 2) in the normal brain structure subgroup. In this subgroup, the plasma levels of decanoylcarnitine, octanoylcarnitine, GPC (15:0/20:4)*, GPC (15:0/22:6)*, and DHEA-S were relatively higher in individuals with epilepsy compared to individuals without epilepsy. On the other hand, the plasma levels of two dihydrosphingomyelins, beta-citrylglutamate, orotidine, N-acetylneuraminate, 3-hydroxy-3-methylglutarate, vanillylmandelate, behenoyl dihydrosphingomyelin (d18:0/22:0)* and aspartate were lower.

The three top-ranking altered metabolites (Supplementary Table S4) all belonged to the dihydrosphingomyelins group, which was interesting, as dihydrosphingomyelins have been shown to create more ordered bilayers in cell membranes than acyl-chain matched sphingomyelins or phosphatidylcholines (29). Dihydrosphingomyelins are important in determining membrane properties, although they are a minor component of cellular sphingolipids (30, 31). Still, their location makes them interesting potential biomarkers compared to previously described amino acids. Interestingly, a study in rat models has shown decreased levels of sphingomyelins in acute epilepsy and elevated levels in the chronic epilepsy group (19). Thus, pointing to the need for further research into the role of sphingomyelins in epilepsy pathogenesis and their potential in treatment.

Beta-citrylglutamate was down-expressed among the epilepsy patients. It is abundant in developing brains and might play a role in activating aconitase in mitochondria as shown in animal models and *in vitro* experiments (32). Low citrate levels, the substrate for synthesizing beta-citrylglutamate, have been frequently observed in patients with epilepsy and epilepsy animal models, as Lai et al. showed in their review (14). Although citrate itself did not reach statistical significance in our cohort, the average z-score in individuals with epilepsy was −0.6, and the TCA cycle may also be affected in the individuals without epilepsy in PED-C as they have other clinical problems. Aconitate (cis/trans) could be another product of citrate that was significantly lower in individuals with epilepsy in the no brain anomalies group.

Another metabolite with low levels was N-acetylneuraminate, also known as sialic acid. It is highly abundant in brain tissue (46). Negatively charged sialic acid units stabilize glycoprotein conformation in cell surface receptors, increasing cell rigidity and enabling signal recognition and adhesion to ligands, antibodies, enzymes, and microbes (25, 33).

Surprisingly, the neurotransmitter aspartate had a significantly lower concentration in the individuals with epilepsy. Previous publications have shown increased levels of aspartate in epilepsy and stroke patients (14).

Analyzing the elderly cohort did not reveal significant changes in lipid metabolism. One possible explanation is the low amount of prevalent cases, with only ~20% of the epilepsy subgroup having received their diagnosis before sample collection. Among participants who received their epilepsy diagnosis after sample collection, the mean time from sample to diagnosis was 9 years (SD = 3.6) in ELD-C, 5.8 years (SD = 2.9) in AD-C, and 1.1 years (SD = 1.4) in PED-C. The changes in metabolism may occur closer to the time of disease onset. Alternatively, the treatment of the disease through medical intervention could affect the metabolic pathways that drive epileptic episodes. This hypothesis is supported by the changes in TAGs that we observed when we analyzed the subgroup of prevalent cases compared to the epilepsy-negative subgroup in ELD-C. Although there was no precise overlap, TAGs were also altered in AD-C, where the percentage of individuals diagnosed before sample collection was much higher (~64%). Alterations in TAGs have been described in animal models and patients with epilepsy (14, 34, 35). For example, a study by Guo et al. compared the metabolic profiles of children with epilepsy who were responsive or resistant to valproic acid treatment and they observed increased TAGs in the non-responder group (36). However, in our study, the subgroups of epilepsy-positive subjects with disease onset before sampling were relatively small in both AD-C and ELD-C. Therefore, further studies in cohorts with more epilepsy-positive cases are needed to confirm this hypothesis.

Comparing the epilepsy incident cases with the epilepsy-negative subgroup in the ELD-C revealed lower C4-OH carnitine and hydrochlorothiazide. C4-OH carnitine is a short-chain acylcarnitine involved in beta-oxidation (37). It can be a product of lipid, amino acid, or carbohydrate metabolism (38). It was altered in both the incident cases and the subgroup with dyslipidemia and epilepsy. However, the subgroups were relatively small, with six subjects belonging to both accounting for two-thirds of the dyslipidemia-positive subgroup and approximately one-third of the incident cases. A higher level of C4-OH carnitine and other short-chain acylcarnitines would be expected in individuals with higher body fat and/or prediabetes (39, 40). Hydrochlorothiazide was also lower in incident cases compared to the epilepsy-negative subgroup of the ELD-C. This is an incidental finding, as hydrochlorothiazide is a diuretic commonly used to treat several diseases, including hypertension (41, 42).

Ketogenic diet (KD), which is a high-fat, low-carbohydrate diet, has been used for treating patients with drug-resistant epilepsy for a long time. It alters lipid levels, and higher concentrations of triglycerides have been reported in patients with KD (43). The exact mechanism of action is still unknown, although, in recent studies, medium-chain triglycerides have been implicated instead of ketones (44).

This study was an exploratory analysis designed to generate hypotheses. Thus, limitations included the following. Firstly, the analyzed cohorts were very different, and there is a possibility of various confounding factors in each cohort. Secondly, although medications may affect the metabolomic profile, we did not have enough data to analyze their effect. The epilepsy-positive subgroups were small and treated with various medications (nutritional and/or medicinal). In the PED-C, we had information about all medicines prescribed to them. However, we only had data about medications belonging to ATC N03A for the AD-C and ELD-C because we did not include any other medication groups in our data request. Still, we do not have a reason to believe that the use of other medications would differ significantly between epilepsy-positive and negative subgroups within one cohort. Also, as dyslipidemia is a common disorder among the adult population (45), we were unable to rule out the presence of undiagnosed cases among the EstBB participants. Conversely, there might be participants with dyslipidemia who use statins or other lipid-modifying agents, and therefore, have normal blood lipid levels. Finally, we must consider that there might be misdiagnosis of epilepsy as well (28).

This study had several strengths as well. Using existing data from the Estonian Biobank and the possibility of returning to the medical records of PED-C during the study period allowed for identifying incident cases of epilepsy who are not receiving treatment yet. Also, to our knowledge, there have not been other studies of this scale that include lipidomics in the untargeted metabolomics analysis of individuals with epilepsy.

## 5 Conclusion

There was no distinct metabolic profile among individuals with epilepsy of different etiology, duration, and type. However, we observed significantly altered metabolites in individuals with epilepsy compared to individuals without epilepsy in a cohort of pediatric patients with normal brain structure, most notably lower levels of dihydrosphingomyelins. The data from EstBB adult participants showed alteration in the triacylglycerol pathway. We believe that untargeted metabolomics can provide valuable insight into epilepsy pathogenesis and potentially improve diagnostics. Further studies in more specific clinical disease groups are needed to confirm this.

## Data Availability

The original contributions of PED-C presented in the study are publicly available. This data can be found here: https://doi.org/10.23673/re-551. The data of AD-C and ELD-C analyzed in this study was obtained from the Estonian Biobank (EstBB). Data access is described in detail on their webpage (https://genomics.ut.ee/en/content/estonian-biobank) and requests should be directed to releases@ut.ee.
